# Human neural stem cells

**DOI:** 10.1111/cpr.13564

**Published:** 2023-10-18

**Authors:** Yu‐Kai Wang, Juan Yu, Ting‐Ting Zhang, Ai‐Jin Ma, Jie Hao, Yue‐Jun Chen, Chang‐Mei Liu, Yan Liu, Chang‐Lin Wang, Pei‐Jun Zhai, Andy Peng Xiang, Tian‐Qing Li, Tie‐Shan Tang, Hong Chen, Xin‐Jie Bao, Yan‐Lin Wang, Wen‐Yan He, Jing Fan, Zhao‐Qian Teng, Liu Wang, Jia‐Xi Zhou, Bo‐Qiang Fu, Yu Vincent Fu, Lin Feng, Jia‐Ni Cao, Ling‐Min Liang, Lei Wang, Qi Zhou, Yu Zhang, Bao‐Yang Hu, Tong‐Biao Zhao

**Affiliations:** ^1^ State Key Laboratory of Stem Cell and Reproductive Biology, Institute of Zoology Chinese Academy of Sciences Beijing China; ^2^ Beijing Institute for Stem Cell and Regenerative Medicine Beijing China; ^3^ Institute for Stem Cell and Regeneration Chinese Academy of Sciences Beijing China; ^4^ National Stem Cell Resource Center Chinese Academy of Sciences Beijing China; ^5^ University of Chinese Academy of Sciences Beijing China; ^6^ Chinese Society for Stem Cell Research Shanghai China; ^7^ Beijing Technology and Business University Beijing China; ^8^ Institute of Neuroscience, Key Laboratory of Neuroscience, CAS Center for Excellence in Brain Science and Intelligence Technology Chinese Academy of Sciences Shanghai China; ^9^ Shanghai Center for Brain Science and Brain‐Inspired Intelligence Technology Shanghai China; ^10^ Institute for Stem Cell and Neural Regeneration, State Key Laboratory of Reproductive Medicine, School of Pharmacy Nanjing Medical University Nanjing China; ^11^ China National Institute of Standardization Beijing China; ^12^ China National Accreditation Service for Conformity Assessment Beijing China; ^13^ Center for Stem Cell Biology and Tissue Engineering, Key Laboratory for Stem Cells and Tissue Engineering, Ministry of Education Sun Yat‐Sen University Guangzhou China; ^14^ State Key Laboratory of Primate Biomedical Research, Institute of Primate Translational Medicine Kunming University of Science and Technology Kunming Yunnan China; ^15^ Yunnan Key Laboratory of Primate Biomedical Research Kunming Yunnan China; ^16^ State Key Laboratory of Membrane Biology, Institute of Zoology University of Chinese Academy of Sciences, Chinese Academy of Sciences Beijing China; ^17^ Department of Rehabilitation Medicine, Tongji Hospital, Tongji Medical College Huazhong University of Science and Technology Wuhan China; ^18^ Department of Neurosurgery, Peking Union Medical College Hospital Chinese Academy of Medical Sciences and Peking Union Medical College Beijing China; ^19^ Department of Neurology The First Affiliated Hospital of Zhengzhou University Zhengzhou China; ^20^ China National Clinical Research Center for Neurological Diseases, Beijing Tiantan Hospital Capital Medical University Beijing China; ^21^ Zhejiang Huode Bioengineering Ltd. Co Hangzhou China; ^22^ Institute of Hematology and Blood Diseases Hospital Chinese Academy of Medical Sciences & Peking Union Medical College Tianjin China; ^23^ National Institute of Metrology Beijing China; ^24^ State Key Laboratory of Microbial Resources, Institute of Microbiology Chinese Academy of Sciences Beijing China; ^25^ Zephyrm Biotechnologies Co., Ltd. Beijing China

## Abstract

‘Human neural stem cells’ jointly drafted and agreed upon by experts from the Chinese Society for Stem Cell Research, is the first guideline for human neural stem cells (hNSCs) in China. This standard specifies the technical requirements, test methods, test regulations, instructions for use, labelling requirements, packaging requirements, storage requirements, transportation requirements and waste disposal requirements for hNSCs, which is applicable to the quality control for hNSCs. It was originally released by the China Society for Cell Biology on 30 August 2022. We hope that publication of the guideline will facilitate institutional establishment, acceptance and execution of proper protocols, and accelerate the international standardization of hNSCs for clinical development and therapeutic applications.

## 1 SCOPE

This document specifies the technical requirements, test methods, test regulations, instructions for use, labelling, packaging, storage, transportation and waste disposal requirements for hNSCs.

This standard is applicable for the quality control of hNSCs derived from primary tissue and differentiated from pluripotent stem cells.

## 2 NORMATIVE REFERENCES

The following content constitute indispensable articles of this standard through normative reference. For dated references, only the edition cited applies. For undated references, only the latest edition (including all amendments) applies.

*Ws 213 Diagnosis of hepatitis C*
*Ws 273 Diagnosis for syphilis*
*Ws 293 Diagnosis for HIV/AIDS*
*T/CSCB 0001 General requirements for stem cells*
*Pharmacopoeia of the People's Republic of China (2020)*
*National Guide to Clinical Laboratory Procedures*

## 3 TERMS, DEFINITIONS AND ABBREVIATIONS

### 3.1 Terms and definitions

The following terms and definitions apply to this document.

#### 3.1.1 Human neural stem cells

Cells in the human central nervous system, which have self‐renewal ability and the potential to differentiate into astrocytes, neurons and oligodendrocytes.

### 3.2 Abbreviations

The following abbreviations are applicable for this document.

EBV: Epstein–Barr virus
HBV: hepatitis B virus
HCV: hepatitis C virus
HCMV: human cytomegalovirus
HIV: human immunodeficiency virus
hNSC: human neural stem cell
HTLV: human T‐lymphotropic virus
STR: short tandem repeats
TP: treponema pallidum

## 4 TECHNICAL REQUIREMENTS

### 4.1 Raw materials and ancillary materials

4.1.1 The collection of raw materials shall be in accordance with the domestically legal and ethical requirements.
4.1.2 The requirements of T/CSCB 0001‐2020 shall be followed.
4.1.3 The donors shall be negative for HIV, HBV, HCV, HTLV, EBV, HCMV and TP.

### 4.2 Primary quality attributes

#### 4.2.1 Cell morphology

In two‐dimensional culture, the nucleus of hNSCs is indistinguishable, cells exhibit a columnar morphology in single‐ or multi‐layers under a microscope. In three‐dimensional culture, cells can form compact and transparent spheres spontaneously.

#### 4.2.2 Chromosome karyotype

The karyotype shall be normal as 46, XX or 46, XY.

#### 4.2.3 Cell viability

The cell viability shall be ≥80% before cryopreservation, and ≥60% post‐thawing.

#### 4.2.4 Cell markers

NESTIN positive rate shall be ≥80.0%, and SOX1/SOX2 double positive rate shall be ≥70.0%.

#### 4.2.5 Characteristics of differentiated cells

hNSCs shall be able to further differentiate into neurons and glial cells (astrocytes and/or oligodendrocytes).

Cell morphology: cells shall contain bipolar or multipolar neurons with long neurites.

Cell markers: cells shall be able to differentiate to GFAP+ and/or S100β+ astrocytes, MAP2+ and/or NeuN+ and/or TUJ1+ neurons, PDGFRα+ and/or A2B5+ and/or O4+ oligodendrocytes.

#### 4.2.6 Microorganisms

Fungi, bacteria, mycoplasma, HIV, HBV, HCV, HTLV, EBV, HCMV and TP shall be negative.

### 4.3 Process control

4.3.1 The process of cell cryopreservation, cell thawing and other cell manufacturing shall follow the requirements of T/CSCB 0001‐2020.
4.3.2 The STR test results of cell products shall be consistent with STR of the donor cell.

## 5 TEST METHODS

### 5.1 Cell morphology

Observe the morphology of cells using an optical microscope.

### 5.2 Chromosome karyotype

The method in the *Pharmacopoeia of the People's Republic of China (2020)* shall be followed.

### 5.3 Cell viability

The method in Annex A shall be followed.

### 5.4 Cell markers

The method in Annex B or C shall be followed.

### 5.5 Microorganisms

#### 5.5.1 Fungi

The ‘1101 Sterility Inspection Method’ in *Pharmacopoeia of the People's Republic of China (2020)* (Volume IV) shall be followed.

#### 5.5.2 Bacteria

The ‘1101 Sterility Inspection Method’ in *Pharmacopoeia of the People's Republic of China (2020)* (Volume IV) shall be followed.

#### 5.5.3 Mycoplasma

The ‘3301 Mycoplasma Inspection Method’ in *Pharmacopoeia of the People's Republic of China (2020)* (Volume IV) shall be followed.

#### 5.5.4 HIV

The nucleic acid test method in WS 293 shall be followed.

#### 5.5.5 HBV

The nucleic acid test method in *National Guide to Clinical Laboratory Procedures* shall be followed.

#### 5.5.6 HCV

The nucleic acid test method in WS 213 shall be followed.

#### 5.5.7 HTLV

The nucleic acid test method in *National Guide to Clinical Laboratory Procedures* shall be followed.

#### 5.5.8 EBV

The nucleic acid test method in *National Guide to Clinical Laboratory Procedures* shall be followed.

#### 5.5.9 HCMV

The nucleic acid test method in *National Guide to Clinical Laboratory Procedures* shall be followed.

#### 5.5.10 TP

The nucleic acid test method in WS 273 shall be followed.

### 5.6 In vitro differentiation capacity

The method in Annex B or C shall be followed.

## 6 INSPECTION RULES

### 6.1 Sampling method

6.1.1 Cells produced from the same production cycle, same production line, same source, same passage and same method are considered to be the same batch.
6.1.2 Three smallest units of packaging shall be randomly sampled from the same batch.

### 6.2 Quality inspection and release

6.2.1 Each batch of products shall be subject to the qualify inspection before release, and inspection reports shall be attached.
6.2.2 The quality inspection items shall include all the attributes specified in 4.2.

### 6.3 Review inspection

Review inspection shall be performed by professional cytological testing institutions or laboratories as necessary.

### 6.4 Decision rules

6.4.1 Products that pass all requirements in 4.2 for the quality inspection for release are considered to be qualified. Products that fail to pass one or more requirements in 4.2 for the quality inspection for release are considered to be unqualified.
6.4.2 Products that pass all requirements in 4.2 for the quality review inspection are considered to be qualified. Products that fail to pass one or more requirements in 4.2 for the review inspection are considered to be unqualified.

## 7 INSTRUCTIONS FOR USAGE

The instructions for usage shall include, but not limited to:

- Product name;
- Passage number;
- Cell number;
- Production date;
- Lot number;
- Production organization;
- Storage conditions;
- Shipping conditions;
- Contact information;
- Operation manual;
- Execution standard number;
- Manufacturing address;
- Postal code;
- Matters that need attention.

*Note*: Provide endotoxin content according to user's requirement.

## 8 LABELS

The label shall include but not limited to:

- Product name;
- Passage number;
- Cell number;
- Lot number;
- Production organization;
- Production date.

## 9 PACKAGE, STORAGE AND TRANSPORTATION

### 9.1 Package

The appropriate materials and containers shall be selected to ensure the maintenance of the primary quality attributes of hNSCs.

### 9.2 Storage

9.2.1 T/CSCB 0001 shall be followed.
9.2.2 Products shall be stored in liquid nitrogen at a temperature below −130°C.

### 9.3 Transportation

9.3.1 T/CSCB 0001‐2020 shall be followed.
9.3.2 Cryopreserved cell products shall be transported in dry ice or below −130°C.

## 10 WASTE DISPOSAL

10.1 Wastes generated during hNSCs production and testing shall follow the waste cell management documents, strictly implement the management standards and make detailed records.
10.2 Any disposal of unqualified cells, leftover cells for disposal or donations during the research and production of hNSCs shall be conducted properly in accordance with appropriate legal and ethical requirements.

## FUNDING INFORMATION

This research is supported by the National Key Research and Development Program of China, Grant/Award Number: 2019YFA0110800, 2019YFA0903800, 2018YFA0108400, 2018YFE0204400, 2021YFA1101600; CAS Project for Young Scientists in Basic Research, Grant/Award Number: YSBR‐041; National Natural Science Foundation of China, Grant/Award Number: 31970821; Joint Funds of the National Natural Science Foundation of China, Grant/Award Number: U21A20396.

## CONFLICT OF INTEREST STATEMENT

No potential conflicts of interest are disclosed.

## ANNEX A

### A.1

(Normative)

Cell viability test (cell enumeration method)

#### A.1. Instruments

A.1.1 Microscope
A.1.2 Haemocytometer

#### A.2. Reagents

Unless otherwise stated, all reagents used shall be analytical grade. The water used for testing shall be deionized water.

A.2.1 Phosphate‐buffered saline: pH 7.4.
A.2.2 Trypan blue solution

#### A.3. Procedure

##### A.3.1. Preparing cell suspension

Harvest and suspend the cells with appropriate volume of phosphate‐buffered saline (A.2.1).

##### A.3.2. Trypan blue staining

Evenly mix the Trypan blue solution (A.2.2) with the cell suspension (A.3.1) at a volume ratio of 1:1.

##### A.3.3. Cell counting

Put coverslips on each chamber of a clean haemocytometer (A.1.2). Transfer 10 μL trypan blue/cell suspension (A.3.2) to the edge of the coverslip, allowing the cell suspension to fully fill the chambers under the coverslip without over‐or underfill. Repeat with the second chamber. Stand for 30 s, count the stained cells and the total cells under microscope (A.1.1), respectively.

For the 16 × 25 counting chamber, use the four 1 mm^2^ medium squares at the top left, top right, bottom left and bottom right of the chamber (i.e., 100 small squares) for counting. For the 25 × 16 counting chamber, use the five 1 mm^2^ medium squares at the top left, top right, bottom left, bottom right and centre of the chamber (i.e., 80 small squares) for counting. When there are cells on the lines of the large square, only cells on the top line and left line of the large square can be counted (or alternatively only cells on the bottom line and right line).

#### A.4. Calculation and analysis

Cell viability is calculated according to Equation (A1):

(A1)
$$S=\left(M-D\right)/M\times 100\%$$

In the equation, *S*—cell viability, *M*—total cell number and *D*—stained cell number.

The viability of cells is the mean of two duplicate samples. Two independent cell viability tests shall be performed on the same sample. The mean value of two independent viability tests is recorded as the viability of cells.

#### A.5. Accuracy

The absolute difference value between the two independent tests, under the same conditions, shall not exceed 10% of their arithmetic mean.

## ANNEX B

### B.1

(Normative)

Detection of cell markers (Immunofluorescent staining method, the arbitral method)

#### B.1. Instruments

Confocal laser scanning microscope

#### B.2. Reagents

B.2.1 Phosphate‐buffered saline (PBS)
B.2.2 4% paraformaldehyde (PFA)
B.2.3 Triton‐X100
B.2.4 Bovine serum albumin (BSA)
B.2.5 Hoechst33342
B.2.6 Anti‐fluorescence quenching agent
B.2.7 Colourless nail polish or coverslip sealant
B.2.8 Antibodies
B.2.9 Prepare the following solutions according to the relative requirements for immunofluorescent staining: blocking/permeabilization solution and antibody dilution solution.

#### B.3. Sample storage

The fixed samples, PBS, 4% PFA and Hoechst33342 shall be stored at 2–8°C. The BSA and anti‐fluorescence quenching agent shall be stored at or below −20°C. Triton‐X100 and colourless nail polish shall be stored at room temperature. Antibodies shall be stored according to the manufacturer's instructions.

#### B.4. Testing protocol

##### B.4.1. Sample preparation and fixation

Put the sterile cover slips in the bottom centre of the cell culture dish and inoculate the cells. When the cells grow to the proper density, discard the culture medium and fix the cells using 4%PFA at room temperature for 15–30 min. Wash the cell samples with PBS for 3 times.

##### B.4.2. Permeabilization and blocking

Permeate and block cells with PBS containing 0.3% Triton X100 and 2% BSA at room temperature for 1–2 h.

##### B.4.3. Antibody incubation

Incubate the cells with the diluted antibodies according to the manufacturer's instructions.

##### B.4.4. Washing

Wash the cells with PBS for 3 times (5–10 min each time).

##### B.4.5. Nuclei staining

Discard PBS, cells are treated with Hoechst33342 solution (diluted 1:1000 in PBS) for 15 min.

##### B.4.6. Coverslips sealing

Add 5 μL of anti‐fluorescence quenching agent to each cover slip, place the cover slip on a microscope slide carefully without forming air bubbles. Seal the edges of coverslips with colourless nail polish or coverslip sealant.

##### B.4.7. Imaging

Choose at least three different visual fields randomly using a 10× or 20× microscope objective for imaging. If there are multiple layers of cells, it is necessary to perform image stacking after multi‐layer scan (interval range 1.5–2.5 μm).

##### B.4.8. Cell counting

Count the Hoechst positive cells in all images, at least 500 cells shall be counted in each image, and the total number shall not be less than 1500 cells. Count the number of antibody positive cells, and calculate the percentage of antibody positive cells.

The percentage of cell marker positive cells is calculated according to Equation (B1):

(B1)
P=B/H×100%

In the equation, *P*—percentage of cell marker positive cells, *B*—antibody positive cells and *H*—Hoechst positive cells, ≥1500.

## ANNEX C

### C.1

(Normative)

Detection of cell markers (Flow cytometry analysis)

#### C.1. INSTRUMENTS

C.1.1 Flow cytometer
C.1.2 Bench‐top centrifuge
C.1.3 Ice maker
C.1.4 Centrifuge tube: 1.5 mL, 50 mL and 15 mL
C.1.5 40 μm cell strainer
C.1.6 Flow tubes

#### C.2. Reagents

C.2.1 Phosphate buffered saline (PBS)
C.2.2 Paraformaldehyde (PFA)
C.2.3 4% paraformaldehyde (PFA)
C.2.4 Antibodies

#### C.3. Sample storage

The fixed samples shall be stored at 2–8°C. The fixing solutions shall be aliquoted, sealed, labelled and stored at or below −20°C. Antibodies shall be stored according to the manufacturer's instructions.

#### C.4. Test protocol

##### C.4.1. Sample preparation

Collect samples by centrifuging single‐cell suspensions with bench‐top centrifuge at 300 *g* for 10 min.

##### C.4.2. Antibody incubation

Incubate the samples with the diluted antibodies according to the manufacturer's instructions. Wash the cell samples with an appropriate volume of wash solution for 2 times, then centrifuge at 300 *g* for 10 min and discard the supernatant.

##### C.4.3. Flow cytometry analysis

Resuspend the samples with wash solution and then transfer the cell suspension into flow cytometry tube by filtering the samples through a 40 μm cell strainer. Load the samples into the flow cytometer and perform testing according to the manufacturer's instruction.

##### C.4.4. Gating

Gate the population of target cells based on particle size and granularity, excluding cell debris and other irrelevant particles. The gating of positive staining cells shall be determined by the fluorescence intensity using isotype controls as a reference. Both positive and negative experimental controls shall be set up for gating and the following analysis.

##### C.4.5. Analysis of results

Analyse the results using software according to manufacturer's instructions.

